# A Case of Fetal Tachycardia after Electroconvulsive Therapy: A Possible Effect of Maternal Hypoxia and Uterine Contractions

**DOI:** 10.1155/2019/3709612

**Published:** 2019-07-04

**Authors:** Anri Watanabe, Nobutaka Ayani, Miyoko Waratani, Tatsuji Hasegawa, Maki Ishii, Teruyuki Matsuoka, Jin Narumoto

**Affiliations:** ^1^Department of Psychiatry, Graduate School of Medical Science, Kyoto Prefectural University of Medicine, Kyoto, Japan; ^2^Department of Obstetrics and Gynecology, Kyoto Prefectural University of Medicine, Graduate School of Medical Science, Kyoto, Japan; ^3^Department of Pediatrics, Graduate School of Medical Science, Kyoto Prefectural University of Medicine, Kyoto, Japan; ^4^Department of Anesthesiology, Graduate School of Medical Science, Kyoto Prefectural University of Medicine, Kyoto, Japan

## Abstract

Electroconvulsive therapy (ECT) is considered to be an effective and safe treatment for depression in pregnant women in that it avoids the risk of psychotropic pharmacotherapy. However, clinicians should be cautious about the adverse effects in the fetus, such as fetal cardiac arrhythmia. Most of the previous studies have demonstrated a reduction in fetal heart rate associated with ECT. However, we encountered a case of fetal tachycardia after maternal ECT-induced convulsions. The patient was a woman who was 30 weeks' pregnant and had severe depression; fetal tachycardia (180–200 bpm) occurred immediately after the electrical stimulation and lasted for more than 30 minutes. The fetal tachycardia might have been caused by maternal hypoxia and uterine contractions. To our knowledge, this is the first report of fetal tachycardia as an adverse effect of ECT. Prolonged fetal tachycardia may cause fetal heart failure. Therefore, oxygenation during convulsions and careful fetal cardiac monitoring are essential when administering ECT in pregnancy.

## 1. Introduction

Pregnant patients with severe psychiatric illnesses, such as affective disorder, are at increased risk of fetal growth restriction, preterm birth, maternal suicide, and infanticide [1–3]. Electroconvulsive therapy (ECT) is considered to be an effective and safe treatment option for patients with antenatal depression during pregnancy because psychotropic drugs can cause teratogenicity and problems in the neonate [4, 5]. Consequently, ECT is considered to be an effective therapy for pregnant patients with psychiatric symptoms [6].

In previous studies and reviews, a reduction in the fetal heart rate (FHR) and arrhythmias have been the most common ECT-related adverse effects in the fetus [6–8] and are attributed to inadequate perfusion of the uteroplacental circulation. Uterine contractions after ECT are often reported during the second and third trimester and might be related to elevated oxytocin [6]. To date, there have been no reports of fetal tachycardia. Here we report a case of fetal tachycardia that might have been caused by maternal hypoxia and uterine contractions in a pregnant woman diagnosed with major depressive disorder (MDD).

## 2. Case Presentation

The patient was a 33-year-old gravida 0 para 0 and had been diagnosed with MDD at the age of 26 years. Although duloxetine had improved her depressive symptoms, she had a relapse of MDD at around 13 weeks' gestation after cessation of duloxetine because of pregnancy at 5 weeks' gestation. Her depressive symptoms then worsened despite resuming duloxetine and she finally attempted suicide. She was admitted to our hospital at 26 weeks' gestation. She did not respond to duloxetine 40 mg/day and experienced loss of appetite and sustained suicidal ideation. She could not tolerate the adverse effects of duloxetine 60 mg/day or aripiprazole 3 mg/day as augmentation therapy (both resulted in headache). A switch to imipramine 25 mg/day caused akathisia, so duloxetine 40 mg/day was resumed because it was tolerated. ECT was then proposed as an adjunctive treatment. After obtaining informed consent from the patient and her family members, we administered a bilateral brief pulse of ECT to achieve a rapid response [9] and improve her suicidal ideation [10].

The patient underwent the first application of ECT at 30 weeks' gestation and FHR was monitored by obstetricians and pediatricians using cardiotocography and fetal echocardiography before and after ECT. ECT was administered twice a week using a Thymatron® System IV machine (Somatics LLC, Lake Bluff, IL, USA). All ECT sessions used an electrical stimulus of 10%–25% to induce the appropriate generalized cerebral seizure with good efficacy. The first and second sessions of ECT caused fetal tachycardia ≥180 bpm for more than 30 minutes (Table 1). At first, we assumed that the first stimulus dose of ECT had been so high that fetal tachycardia had occurred, so the stimulus dose was lowered in the second session. Nevertheless, fetal tachycardia occurred during the second session of ECT; we considered that the length of maternal apnea affected the fetal cardiac symptoms, so we started oxygenation just after the electric stimulus. The fetus did not have persistent elevation of FHR in the third, fourth, and fifth sessions of ECT. Meanwhile, cardiotocography recorded uterine contractions after ECT from the third session onward. On the sixth session of ECT, FHR monitoring showed tachycardia for around 120 minutes again. During monitoring, though cardiotocography did not show acceleration, baseline variability was within normal limits and decelerations were not seen. The patient had a partial remission (a change in her Hamilton Rating Scale for Depression-24 score from 36 to 26 points) with recovery of appetite and relief of suicidal ideation after the 6th session of ECT. We were concerned about the risk of preterm labor caused by administration of ECT and decided to terminate ECT because the uterine contractions had been longer at the most recent session (lasting around 120 minutes) than previously, and the risks of continuing ECT by then outweighed the benefits.

The patient was discharged at 34 weeks' gestation and delivered a healthy baby (female, weighed 2,812 g, Apgar score of 9/10 at 1/5 minutes) at 38 weeks' gestation. The infant has been followed up by the Department of Pediatrics at our hospital for two years and the patient has achieved a complete remission.

## 3. Discussion

We have encountered a rare case of persistent fetal tachycardia (180–200 bpm for over 30 minutes), although reduction of FHR, including bradycardia, is a common side effect of maternal ECT [6]. Fetal tachycardia is defined as a heart rate over 160 bpm and is observed during fetal mild hypoxia, recovery from a hypoxygenic state, infection in utero, and maternal medical conditions (e.g., hyperthyroidism or maternal infection) and may be associated with medication (beta-agonists) [11]. The result of the blood tests showed no thyroid dysfunction or infection, and the patient did not take any drugs that might result in fetal tachycardia. In the present case, we considered the elevation of FHR might be caused by a long duration of maternal apnea after ECT that led to mild fetal hypoxia because the start of oxygenation was delayed after the convulsions following the first and second electric shocks. Therefore, restarting oxygenation just after the electric shock could avoid persistent fetal tachycardia from the third to fifth ECT session.

Uterine contractions occurred after the third ECT session in this case. Uterine contractions are common adverse effects of ECT during the third trimester [6, 8, 12]. Tocolytic agents are sometimes used to suppress contractions [12–14] but were not used in our patient because the duration of uterine contractions was short (10–20 minutes). However, on the last session of ECT, the patient experienced prolonged uterine contractions lasting for around 120 minutes. Uterine contractions are believed to reduce placental blood flow [15], which lead to fetal hypoxia. In our case, the baseline variability was within normal limits, indicating that the fetus had not developed acidosis. Therefore, we hypothesize that the sustained uterine contractions induced by ECT caused the fetal tachycardia with mild fetal hypoxia.

As far as we know, this is the first case report of fetal tachycardia caused by maternal ECT. Clinicians tend to be more interested in fetal bradycardia than in fetal tachycardia because the latter is usually less troublesome. Nevertheless, it is still important to be aware of tachycardia. Tachyarrhythmias, including tachycardia, are mainly caused by maternal infection or fetal hypoxia and can be lethal despite specific treatments [16, 17]; sustained fetal tachycardia might lead to fetal heart failure, hydrops, and polyhydramnios [11].

In conclusion, we have encountered a case of fetal tachycardia after ECT that was related to the duration of the fetal hypoxia induced by maternal hypoxia or uterine contractions. ECT can affect the cardiovascular system so it should be administered with careful monitoring of FHR not only for bradycardia but also for tachycardia during pregnancy. Restarting oxygenation immediately after delivery of the electric shock, especially, is important to avoid maternal apnea which leads to persistent fetal tachycardia.

## Figures and Tables

**Table 1:** Results of fetal heart rate monitoring and duration of maternal apnea at each ECT session.

ECT	Stimulus (%)	Seizure duration, seconds	FHR, bpm	Duration of tachycardia, minutes	Uterine contraction	Maternal apnea, seconds
1	15	93	180–190	60	No	110
2	10	69	190–200	30	No	80
3	15	100	150	N/A	Yes (20 minutes)	20
4	25	25	180–190	Several	Yes (15 minutes)	20
5	25	38	150	N/A	Yes (10 minutes)	20
6	25	73	180–190	Around 120	Yes (around 120 minutes)	20

All values except for seizure duration are approximate. ECT, electroconvulsive therapy; FHR, fetal heart rate; N/A, not applicable.

## References

[1] Männistö T., Mendola P., Kiely M. (2016). Maternal psychiatric disorders and risk of preterm birth. *Annals of Epidemiology*.

[2] Lysell H., Dahlin M., Viktorin A. (2018). Maternal suicide – Register based study of all suicides occurring after delivery in Sweden 1974–2009. *PLoS ONE*.

[3] Flynn S. M., Shaw J. J., Abel K. M. (2013). Filicide: mental illness in those who kill their children. *PLoS ONE*.

[4] Liu D., Xu P., Jiang K. (2017). The use of psychotropic drugs during pregnancy. *Shanghai Archives of Psychiatry*.

[5] McAllister-Williams R. H., Baldwin D. S., Cantwell R. (2017). British Association for Psychopharmacology consensus guidance on the use of psychotropic medication preconception, in pregnancy and postpartum 2017. *Journal of Psychopharmacology*.

[6] Anderson E. L., Reti I. M. (2009). ECT in pregnancy: a review of the literature from 1941 to 2007. *Psychosomatic Medicine*.

[7] DeBattista C., Cochran M., Barry J. J., Brock-Utne J. G. (2003). Fetal heart rate decelerations during ECT-induced seizures: is it important?. *Acta Anaesthesiologica Scandinavica*.

[8] Leiknes K. A., Cooke M. J., Jarosch-von Schweder L., Harboe I., Høie B. (2015). Electroconvulsive therapy during pregnancy: a systematic review of case studies. *Archives of Women's Mental Health*.

[9] Sackeim H. A., Prudic J., Devanand D. P. (2000). A prospective, randomized, double-blind comparison of bilateral and right unilateral electroconvulsive therapy at different stimulus intensities. *Archives of General Psychiatry*.

[10] Kellner C. H., Greenberg R. M., Murrough J. W., Bryson E. O., Briggs M. C., Pasculli R. M. (2012). ECT in treatment-resistant depression. *The American Journal of Psychiatry*.

[11] Bravo-Valenzuela N., Rocha L., Machado Nardozza L., Júnior E. (2018). Fetal cardiac arrhythmias: Current evidence. *Annals of Pediatric Cardiology*.

[12] Walker R., Swartz C. M. (1994). Electroconvulsive therapy during high-risk pregnancy. *General Hospital Psychiatry*.

[13] Bhatia S. C., Baldwin S. A., Bhatia S. K. (1999). Electroconvulsive therapy during the third trimester of pregnancy. *Journal of ECT*.

[14] Ishikawa T., Kawahara S., Saito T. (2001). Anesthesia for electroconvulsive therapy during pregnancy -a case report. *Masui*.

[15] Sato M., Noguchi J., Mashima M., Tanaka H., Hata T. (2016). 3D power Doppler ultrasound assessment of placental perfusion during uterine contraction in labor. *Placenta*.

[16] Singh G. K. (2004). Management of fetal tachyarrhythmias. *Current Treatment Options in Cardiovascular Medicine*.

[17] Wacker-Gussmann A., Strasburger J., Cuneo B., Wakai R. (2014). Diagnosis and treatment of fetal arrhythmia. *American Journal of Perinatology*.

